# Advances in Molecular Understanding of Ocular Adnexal Disease

**DOI:** 10.3390/ijms25136896

**Published:** 2024-06-24

**Authors:** Robert M. Verdijk

**Affiliations:** 1 Department of Pathology, Section Ophthalmic Pathology, Erasmus University Medical Center, Doctor Molewaterplein 40, 3015 GD Rotterdam, The Netherlands; r.verdijk@erasmusmc.nl; 2 Department of Ocular Oncology, The Rotterdam Eye Hospital, Schiedamse Vest 180, 3011 BH Rotterdam, The Netherlands; 3 Department of Pathology, Leiden University Medical Center, Albinusdreef 2, 2333 ZA Leiden, The Netherlands

The goal of this Special Issue is to provide comprehensive molecular biological data that aims to elucidate the molecular and epigenetic mechanisms operable in diseases of the ocular adnexa. As rare as these may be, a better understanding of their molecular genetic bases and epigenetics is essential for improving the diagnosis and treatment of patients. Detailed information on chromosomal aberrations, gene fusions, mutations, and epigenetics offers important new insights. The manuscripts submitted for this Special Issue come from research groups in China and Europe and cover diseases as diverse as degenerative and neoplastic. The data presented come both from mouse models and patients and have been either newly generated, obtained from open sources, or presented in the form of an extensive literature review.

Two studies from China have utilized mouse models in order to elucidate the mechanisms underlying retinitis pigmentosa. Zhou et al. (2023) used spatial transcriptomic analysis to study the changes in different retinal layers in the well-known rd1 mouse model at different ages. This demonstrates photoreceptor apoptosis in both the early and late stages of retinitis pigmentosa. In the anterior layers of the retina, neuronal apoptosis, positive regulation of synaptic transmission, and ATP metabolic process are upregulated, whereas in the posterior layers, upregulated genes are mainly involved in visual perception and visual phototransduction. Over time, differentially expressed genes are mainly involved in the ATP metabolic process and energy derivation by the oxidation of organic compounds. Enhanced oxidative stress in the late stage of retinitis pigmentosa is accompanied by an upregulation of the VEGF pathway. Spatial transcriptomic analysis with a precise sampling method thus identified patterns of the changes in key pathways in the early and late stages of retinitis pigmentosa in this mouse model. Chen et al. (2023) detailed the development of a Cep250 KO mouse model to investigate the pathogenesis of Usher syndrome. OCT at P180 showed the thickness of the ONL, IS/OS, and whole retina of Cep250 mice to be significantly reduced. The a-wave and b-wave amplitudes of Cep250 mice in scotopic and photopic ERG were lower, and histology showed reduced photoreceptors through apoptosis. RNA-seq analysis showed that the cGMP/PKG signaling pathways, MAPK signaling pathways, edn2/fgf2 axis pathways, and thyroid hormone synthesis were upregulated, whereas protein processing in the endoplasmic reticulum was downregulated in Cep250 KO eyes. The dysregulation of the cGMP/PKG/MAPK pathways, well described in neoplastic diseases, may also contribute to the pathogenesis of cilia-related retinal degeneration.

The MAPK pathway is recurrently activated through driver mutations in *BRAF*, *NRAS*, or *NF1* in conjunctival melanoma. These mutations, however, are of limited use for prognosis in conjunctival melanoma. Van Ipenburg et al. (2023) followed up on their earlier investigations about the prognostic importance of telomerase upregulation in conjunctival melanoma and the risk of metastasis [1]. The *TERT* promoter mutation, which is present in 43% of conjunctival melanomas, is associated with reduced metastasis-free survival [2]. Enhanced telomere maintenance is a key event in cellular immortality in order to escape cellular crises, potentially occurring through activation of telomerase (TERT) or through alternative lengthening of telomeres (ALT), most commonly resulting from ATRX inactivation. ATRX loss and *TERT* promoter mutations were only found in pre-malignant conjunctival melanocytic lesions, adding ATRX immunohistochemistry to the diagnostic toolbox for identifying malignant melanocytic lesions in the conjunctiva that may be potentially correlated to an adverse outcome.

Reggiani et al. (2023, 2024) showed in two separate studies how publicly available data may be used to answer newly formulated research questions. They evaluated the interdependence of prognostic molecular genetic features in uveal melanoma. Reanalysis of data from three separate public datasets confirmed that *BAP1* mutations are always associated with monosomy of chromosome 3 in high-risk patients. Although other features (6p gain, 8q gain, monosomy 3, and *SF3B1* mutation) were present independently from each other, gain of 8q is mainly associated with monosomy 3, and gain of 8q is seen often in combination with gain of 6p and *SF3B1* mutations. The group at high risk for metastasis contained patients with 8q gain–monosomy 3 or *BAP1* mutation–monosomy 3. On the other hand, patients with 6p gain combined with either 8q gain or *SF3B1* mutations were mainly represented in the low-risk cluster. This last finding indicates that the results from public data are as reliable as the scope and accuracy of that dataset. The median follow-up for the Genoa dataset (n = 33) was 2 months, and for the TCGA dataset (n = 80), it was 27 months. Cohorts with a more extended follow-up also revealed the recurrent combination of *SF3B1* mutations with the gain of chromosomes 6 and 8 [3]. However, these patients have been shown to be at intermediate risk for metastasis, which may occur much later than the median follow-up period provided in the studies reanalyzed by Reggiani et al. [4,5]. The use of public datasets for new research questions is to be recommended, as the valuable clinical data derived from patients may, in the long run, offer new insights and a better understanding and treatment of the disease for many more patients in the future. However, such a “living” dataset would greatly benefit from regular updates on the clinical follow-up of the original cohort and thus prove to be even more prospectively usable for the benefit of all. In the second study, Reggiano et al. (2024) compared different machine learning methods to detect relevant genes for metastatic disease prediction in the TCGA uveal melanoma dataset. Detected targets were validated with multi-gene score analysis on a larger uveal melanoma microarray dataset published by the same group and then used for gene selection in uveal melanoma [6]. In this study, it is explicitly stated by the authors that metastases can also develop after the end of follow-up. Indeed, high-risk cases that did not develop metastases during follow-up might do so afterwards. In their work, these authors have shown that data fusion can potentially improve patient classification, as two patients previously classified by single domain analysis as high-risk but who had not developed metastasis during follow-up were now reclassified as low- and intermediate-risk patients when the additional data were taken into consideration. This shows that the use of multiple datasets to evaluate prognostic methods is essential to obtaining an accurate estimate of the reliability of such a classification method. Some genes could be affected by multiple genomic events that inactivate their expression. Single domain analysis failed to detect these genes as significantly altered in tumors, while the analysis of multiple domains could be a strong basis to distinguish between genes with a functional role in pathogenesis and those not causally involved markers.

Dahl Vest et al. (2024) investigated the genetic profile of 26 patients with diffuse large B-cell lymphoma, not otherwise specified (DLBCL-NOS), and two patients with high-grade B-cell lymphoma (HGBCL) with *MYC* and *BCL2* rearrangements presenting in the ocular adnexa. Primary ocular adnexal DLBCL-NOS was generally characterized by non-germinal center B-cell origin and LymphGen MCD subtype. Primary ocular adnexal DLBCL-NOS presented pathogenic variants in genes involved in NF-κB activation and genes that are recurrently mutated in other extranodal lymphomas of non-GCB origin, including *MYD88* (29%), *CD79B* (21%), *PIM1* (21%), and *TBL1XR1* (21%). Relapsed DLBCL-NOS presenting in the ocular adnexa showed a similar pattern. This contribution provides the most comprehensive genetic study of DLBCL-NOS presenting in the ocular adnexa and the first study to elucidate genetic variants in ocular adnexal HGBCL. Incorporation of genetic tumor profiling holds promise to be the next step in future precision medicine with the selection of targeted therapy based on specific genetic subtypes and involved biological pathways, although results so far have been rather disappointing [7].

Powell et al. (2023) provide a comprehensive review of the molecular landscape of adenoid cystic carcinoma, the most frequent malignant tumor in the lacrimal gland. This review detailed recurrent *MYB*–*NFIB* chromosomal translocations (50%), *NOTCH1* and *NOTCH2* mutations (30%), and Notch signaling pathway overexpression (65%). There is a lack of data on the role of DNA damage repair gene mutations in adenoid cystic carcinoma of the lacrimal gland. Epigenetic modifications in adenoid cystic carcinoma of the lacrimal gland target the chromatin-remodeling genes *SMARCA2*, *KDM6A*, and *CREBBP*. These findings are comparable to those of salivary gland tumors. Whilst clinical trials aimed at inhibiting Notch have shown initial promise in salivary gland adenoid cystic carcinoma, it is of paramount importance that further large-scale studies aimed at targeting the underlying cellular mechanisms and biological processes that govern adenoid cystic carcinoma of the lacrimal gland are conducted. The development of targeted therapeutic options to ameliorate disease will pave the way for personalized therapy, ultimately improving overall patient survival rates and reducing morbidity and mortality associated with adenoid cystic carcinoma of the lacrimal gland.

The editor wishes to thank all authors for their contribution to this Special Issue, “Advances in Molecular Understanding of Ocular Adnexal Disease”. Moreover, they also particularly wish to extend their appreciation to the members of the Asia-Pacific Academy of Ophthalmology, the European Ophthalmic Pathology Society, and the Ophthalmic Oncology Group. It is through such professional networks that many of us are introduced to the most recent developments in this field and stimulate each other to collaborate and advance science and, ultimately, patient care.
